# Latent class analysis to define radiological subgroups in pulmonary nontuberculous mycobacterial disease

**DOI:** 10.1186/s12890-018-0675-8

**Published:** 2018-08-31

**Authors:** Steven A. Cowman, Joseph Jacob, Sayed Obaidee, R. Andres Floto, Robert Wilson, Charles S. Haworth, Michael R. Loebinger

**Affiliations:** 1 National Heart and Lung Institute, Imperial College London, London, UK; 2 Host Defence Unit, Royal Brompton Hospital, London, UK; 3 Department of Radiology, Royal Brompton Hospital, London, UK; 4 Cambridge Centre for Lung Infection, Papworth Hospital, Cambridge, UK; 5 Department of Medicine, University of Cambridge, Cambridge, UK

**Keywords:** Nontuberculous mycobacteria, Latent class analysis, High resolution computed tomography

## Abstract

**Background:**

Nontuberculous mycobacterial (NTM) pulmonary disease has conventionally been classified on the basis of radiology into fibrocavitary and nodular-bronchiectatic disease. Whilst being of great clinical utility, this may not capture the full spectrum of radiological appearances present. The aim of this study was to use latent class analysis (LCA) as an unbiased method of grouping subjects with NTM-pulmonary disease based on their CT features and to compare the clinical characteristics of these groups.

**Methods:**

Individuals with NTM-pulmonary disease were recruited and a contemporaneous CT scan obtained. This was scored using an NTM-specific scoring system. LCA was used to identify groups with common radiological characteristics. The analysis was then repeated in an independent cohort.

**Results:**

Three classes were identified in the initial cohort of 85 subjects. Group 1 was characterised by severe bronchiectasis, cavitation and aspergillomas, Group 2 by relatively minor radiological changes, and Group 3 by predominantly bronchiectasis only. These findings were reproduced in an independent cohort of 62 subjects. Subjects in Group 1 had a lower BMI and serum albumin, higher serum CRP, and a higher mortality.

**Conclusions:**

These findings suggest that NTM-pulmonary may be divided into three radiological subgroups, and that important clinical and survival differences exist between these groups.

**Electronic supplementary material:**

The online version of this article (10.1186/s12890-018-0675-8) contains supplementary material, which is available to authorized users.

## Background

Pulmonary non-tuberculous mycobacterial disease is a challenging infection which is associated with a high mortality [1–3]. Treatment is frequently poorly tolerated, expensive, and in many cases response rates are poor [4]. Radiology is essential for the diagnosis of disease and has important implications in guiding treatment. NTM-pulmonary disease has been observed to commonly fall into two clinico-radiological patterns of disease, fibrocavitary and nodular-bronchiectatic disease, and this classification forms the basis of treatment recommendations in guidelines [5] and has important prognostic implications [6–8]. Whilst being of great clinical utility this division may not capture the spectrum of radiological appearances, some of which may not fit clearly into either group [3, 9–11].

The aim of this study was to characterise the radiological features of NTM-pulmonary disease using an NTM-specific scoring system, to use latent class analysis (LCA) as an unbiased method to identify subgroups sharing common patterns of radiological features, and to examine the clinical characteristics associated with any such patterns.

## Methods

Individuals with NTM-pulmonary disease were recruited prospectively from the outpatient department of the Royal Brompton Hospital and Chelsea and Westminster Hospital between September 2012 and November 2013. All met American Thoracic Society 2007 disease criteria [5]. Subjects were excluded if they had a diagnosis of cystic fibrosis, HIV infection or other primary or secondary immunodeficiency or if they were receiving any immunosuppressant medication other than oral prednisolone. A HRCT scan of the thorax was performed in subjects who had not undergone such a scan in the previous 6 months. In those who declined additional imaging as part of the study their most recent clinical HRCT was obtained. Subjects also underwent a full clinical assessment, blood and sputum sampling, St George’s Respiratory Questionnaire (SGRQ) and lung function testing. Written consent was gained from all participants and the study was approved by the local Research Ethics Committee (reference 12/LO/1034). Further details are provided in the supplementary materials.

CT scoring was performed as previously described [12] by a specialist radiologist with 5 years of experience in thoracic imaging, blinded to clinical details. For bronchiectasis extent, bronchiectasis severity, tree-in-bud opacification, nodules and consolidation, tertiles of the maximum possible score for each feature were used to categorise the features as low, medium or high. Cavitating nodules, severe cavitation and aspergilloma scores were dichotomised as present or absent. As a measure of the overall severity of an individual’s radiological disease burden, composite CT scores were calculated for each participant by expressing the individual scores for bronchiectasis extent, bronchiectasis severity, tree-in-bud opacification, nodules, consolidation, cavitating nodules, severe cavitation and aspergilloma as a percentage of the maximum possible score for that feature, then summing the scores together. The scoring proforma is detailed in Additional file 1: Table S1 of the supplementary material.

Statistical analysis was performed in the R environment version 3.4.0 [13]. Latent class analysis (LCA) was performed on the matrix of CT scores using the fpc and flexmix packages [14, 15]. LCA is a statistical method which takes a set of multivariate data and uses this to identify groups of related subjects (‘latent classes’) within the data which share similar characteristics. The Aikake information criteria (AIC) was used to identify the optimum number of classes in the model as the number giving the lowest value of AIC [16]. Composite CT scores were compared between groups using analysis of variance (ANOVA). Clinical characteristics were compared between groups using ANOVA, Tukey’s Honestly Significant Difference test and Fisher’s exact test. Survival curves were generated using the survival package [17] and compared using the Log-rank test. Correction for multiple testing was performed using the Benjamini-Hochberg method.

To examine the reproducibility of the groups identified by LCA and their radiological characteristics, the analysis was repeated on an independent cohort from another tertiary centre. Subjects with NTM-pulmonary disease at Papworth Hospital who underwent CT scanning at as part of their clinical care were identified retrospectively, their anonymised imaging and clinical data were retrieved and data analysed using the exact methodology used for the original cohort.

## Results

A total of 85 subjects were recruited, their clinical and radiological characteristics are shown in Additional file 1: Tables S2 and S3 of the supplementary material. Twelve subjects declined further imaging but had undergone HRCT for clinical purposes which were obtained for analysis. The median interval between imaging and recruitment was 23 days. In 80 (94%) subjects a HRCT was obtained within twelve months of recruitment.

### Identification of latent classes

**Fig. 1:**
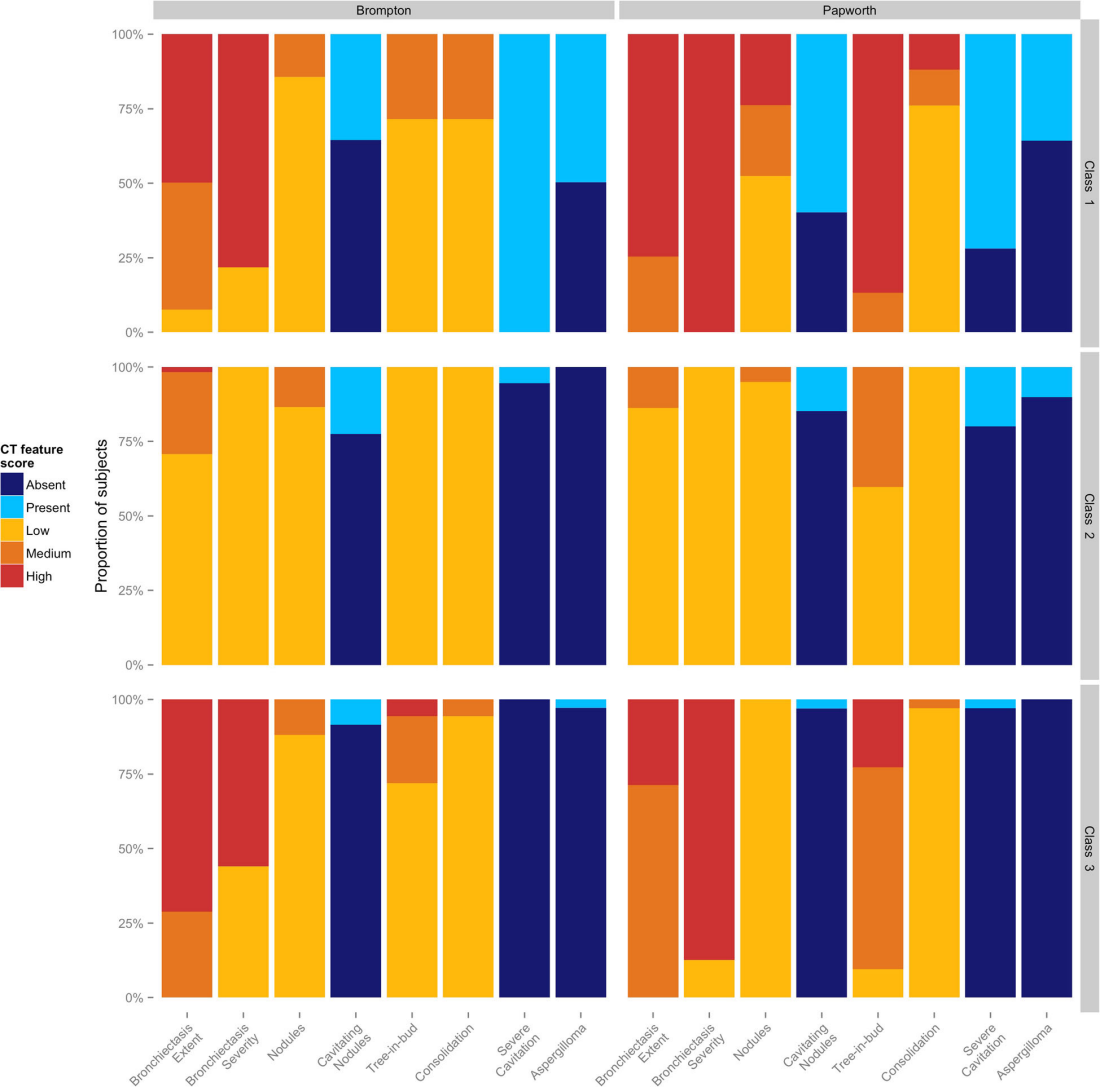
CT features of latent classes identified in the Royal Brompton (left panel) and Papworth (right panel) cohorts. The three classes identified by latent class analysis in each cohort are shown in the top, middle and bottom panels. The individual CT features used in the NTM scoring system are shown on the x-axes and the proportion of subjects on the y-axes. Colours represent the severity (low, medium or high), or the presence or absence of the CT feature

**Fig. 2:**
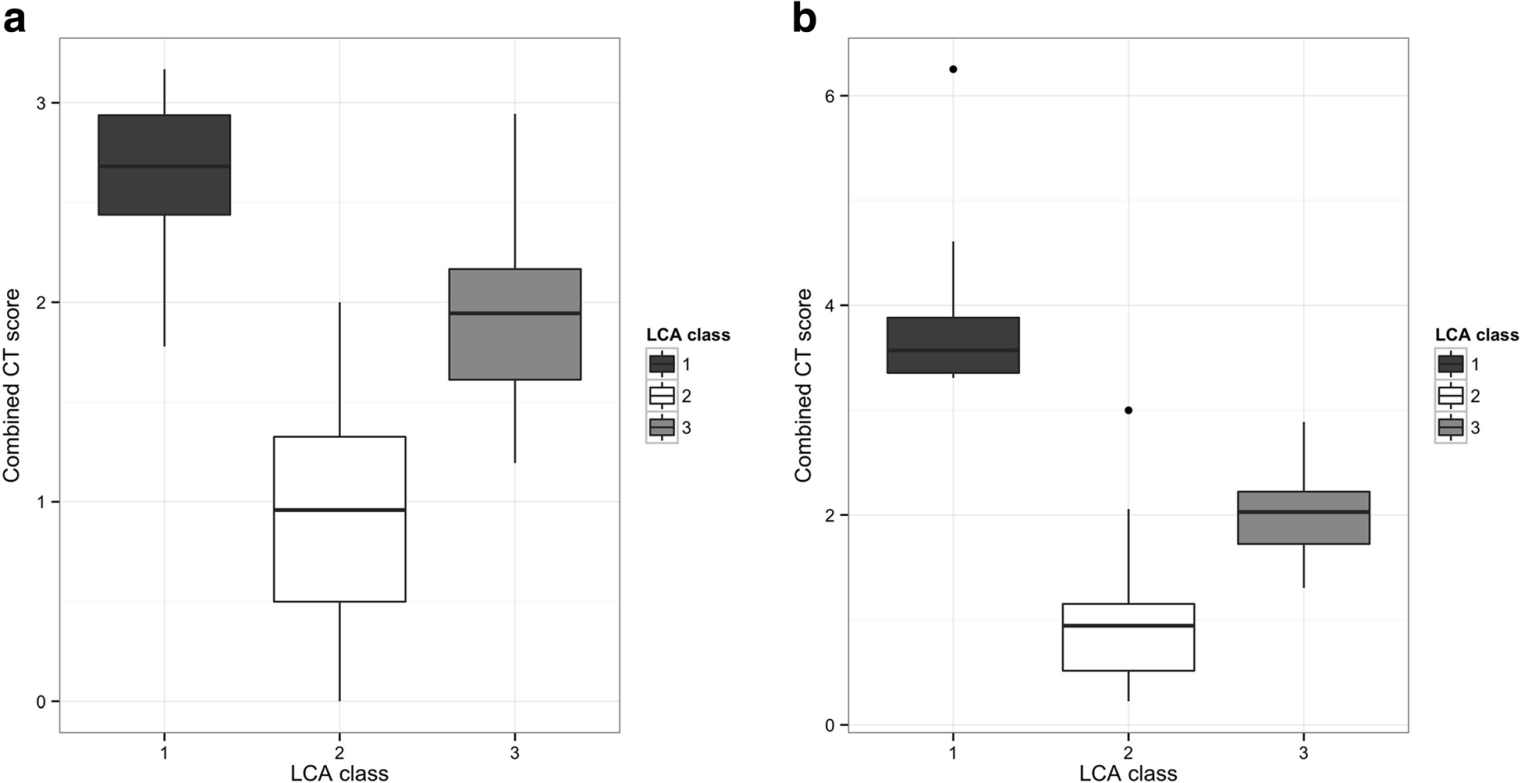
Composite CT scores between latent classes in the a) Royal Brompton and b) Papworth cohorts. The three classes identified by latent class analysis in each cohort are shown on the x-axes, the composite CT score for each class are shown on the y-axis

Three latent classes were identified using the AIC (see Additional file 1: Figure S1). There were 14 subjects (16.4%) in class 1, 38 (44.7%) in class 2 and 33 (38.8%) in class 3. The individual CT features of each of the latent classes are shown in Fig. 1 and the total composite CT scores in each class are shown in Fig. 2a.

The first class, referred to as “Cavitary” disease, was characterised by the universal presence of severe cavitation with medium or highly extensive bronchiectasis. There was a high incidence of aspergilloma in this group, seen in almost half, whereas they were almost absent from the other groups. The second class, referred to as “Nodular” disease had the lowest composite CT scores and a relative lack of radiological changes, with the exception of nodules (medium levels in 13.5%) and cavitating nodules which were present in 22.5%. The third class, referred to as “Bronchiectatic” disease had the most extensive bronchiectasis (high in 71.2%) which was of high severity in the majority (56%), and the highest frequency of tree-in-bud changes (medium or high levels in 28%), although cavitation and nodules were uncommon.

The composite CT score (Fig. 2a) was highest in Cavitary disease (mean 2.62), intermediate in Bronchiectatic disease (mean 1.96) and lowest in Nodular disease (0.95). All differences were significant at *P* < 0.001.

### Clinical differences

The clinical characteristics of the latent classes are shown in Table 1. Subjects from the Cavitary group were significantly older than those in other classes (*P* = 0.003 vs Nodular, *P* = 0.079 vs Bronchiectatic groups). They had a higher prevalence of semi-invasive aspergillosis, were more likely to have received treatment for NTM and over one-third were receiving systemic corticosteroids, although these differences were not significant. The mean C-reactive protein level was higher than both other classes (*P* < 0.001 vs Nodular, *P* = 0.002 vs Bronchiectatic groups), body mass index was lower (*P* = 0.003 vs Nodular, *P* = 0.008 vs Bronchiectatic groups) and serum albumin was lower than in the Nodular (*P* = 0.007) but not the Bronchiectatic (*P* = 0.246) group. The mean corrected carbon monoxide transfer factor (TLCOc) was the lowest of the three groups, but this did not reach significance. Dyspnoea and quality of life scores were not significantly worse than the other two classes.

**Table 1:** Clinical characteristics of latent classes in the Royal Brompton cohort

	Class			*P* value	Adjusted *P* value
	1	2	3		
	*N* = 14	*N* = 38	*N* = 33		
Female Sex	9 (64.3%)	22 (57.9%)	23 (69.7%)	0.571	0.670
Age (years)	72.4 (±10.0)	61.5 (±11.1)	66.8 (±9.1)	**0.003**	**0.037**
Smoking				0.411	0.575
Never smoker	6 (42.9%)	18 (47.4%)	17 (51.5%)		
Ex-smoker	5 (35.7%)	16 (42.1%)	15 (45.5%)		
Current smoker	3 (21.4%)	4 (10.5%)	1 (3.0%)		
White - British Ethnicity	12 (85.7%)	30 (78.9%)	28 (84.8%)	0.747	0.747
Diagnosis				**0.006**	**0.039**
Bronchiectasis	5 (35.7%)	13 (34.2%)	25 (75.8%)		
COPD	5 (35.7%)	11 (28.9%)	4 (12.1%)		
Other	0 (0.0%)	6 (15.8%)	2 (6.1%)		
No underlying disease	4 (28.6%)	8 (21.1%)	2 (6.1%)		
Systemic corticosteroids	4 (28.6%)	7 (18.4%)	5 (15.2%)	0.574	0.670
Oral antibiotic prophylaxis	2 (14.3%)	9 (23.7%)	11 (33.3%)	0.404	0.575
Nebulised antibiotic prophylaxis	1 (7.1%)	2 (5.3%)	4 (12.1%)	0.677	0.702
Chronic *Pseudomonas* infection	0 (0.0%)	3 (7.9%)	7 (21.2%)	0.088	0.176
Semi-invasive Aspergillosis	2 (14.3%)	1 (2.6%)	0	0.069	0.155
NTM species				0.612	0.685
*M. abscessus*	4 (28.6%)	4 (10.5%)	6 (18.2%)		
*M. avium* complex	7 (50.0%)	18 (47.4%)	19 (57.6%)		
*M. kansasii*	1 (7.1%)	4 (10.5%)	4 (12.1%)		
*M. xenopi*	1 (7.1%)	8 (21.1%)	2 (6.1%)		
Other species	1 (7.1%)	4 (10.5%)	2 (6.1%)		
Sputum smear positive	1 (7.1%)	3 (7.9%)	5 (15.2%)	0.639	0.688
Currently receiving NTM treatment	6 (42.9%)	8 (21.1%)	4 (12.1%)	0.072	0.155
Ever received NTM treatment	11 (78.6%)	15 (39.5%)	11 (33.3%)	**0.015**	0.060
Duration of NTM disease (years)	3.4 (±3.2)	1.6 (±2.6)	4.3 (±4.7)	**0.01**	**0.047**
Age at diagnosis (years)	69.6 (±9.9)	60.0 (±11.5)	62.5 (±10.6)	**0.035**	0.098
BMI (kg/m^2^)	18.3 (±3.2)	22.6 (±4.3)	22.3 (±3.4)	**0.004**	**0.037**
SGRQ total score	51.3 (±24.6)	42.1 (±26.1)	48.1 (±21.6)	0.443	0.591
MRC dyspnoea score	2.9 (±1.4)	2.4 (±1.5)	2.6 (±1.1)	0.513	0.653
FEV1 (% predicted)	58.7 (±13.3)	69.5 (±27.6)	53.9 (±20.1)	**0.024**	0.081
FVC (% predicted)	83.1 (±27.0)	97.7 (±20.8)	85.8 (±16.8)	**0.026**	0.081
TLCOc (% predicted)	47.9 (±24.1)	62.2 (±26.0)	59.2 (±18.2)	0.25	0.389
Haemoglobin (g/dL)	12.7 (±1.7)	13.7 (±1.6)	13.7 (±1.1)	**0.049**	0.125
Neutrophil count (× 10^9^/L)	7.0 (±2.9)	5.3 (±3.0)	6.1 (±3.1)	0.186	0.326
Lymphocyte count (× 10^9^/L)	1.4 (±0.5)	1.6 (±0.6)	1.8 (±0.7)	0.242	0.389
Serum albumin (g/L)	36.0 (±6.3)	40.2 (±3.7)	38.2 (±3.9)	**0.007**	**0.039**
CRP (mg/L)	37.2 (±51.1)	7.1 (±13.7)	10.0 (±13.5)	**< 0.001**	**0.017**
Platelet count (× 10^9^/L)	298 (±126)	244 (±62)	255 (±86)	0.126	0.235

Continuous variables are given as mean ± standard deviation and compared using ANOVA, categorical values are given as number and percentage and compared using Fisher’s exact test

*COPD* chronic obstructive pulmonary disease, *BMI* body mass index, *SGRQ* St George’s Respiratory Questionnaire, *MRC* Medical Research Council dyspnoea scale, *FEV1* forced expiratory volume in 1 s, *FVC* forced vital capacity, *TLCOc* corrected transfer factor for carbon monoxide, *CRP* C-reactive protein

Values shown in bold indicate *P* < 0.05

Compared to the other groups, the Nodular group had a more even mix of underlying respiratory pathology and 21% had no underlying disease. Lung function indices were the highest of the three groups. This group had the shortest mean duration of NTM disease prior to enrolment (*P* = 0.307 vs Cavitary, *P* = 0.008 vs Bronchiectatic groups).

Significantly more subjects (75.8%) in the Bronchiectatic group had previously diagnosed underlying bronchiectasis and 21.2% were chronically infected with Pseudomonas, which was uncommon in the other groups. The Bronchiectatic group had the highest rates of antibiotic prophylaxis and the lowest rate of current NTM treatment.

**Fig. 3:**
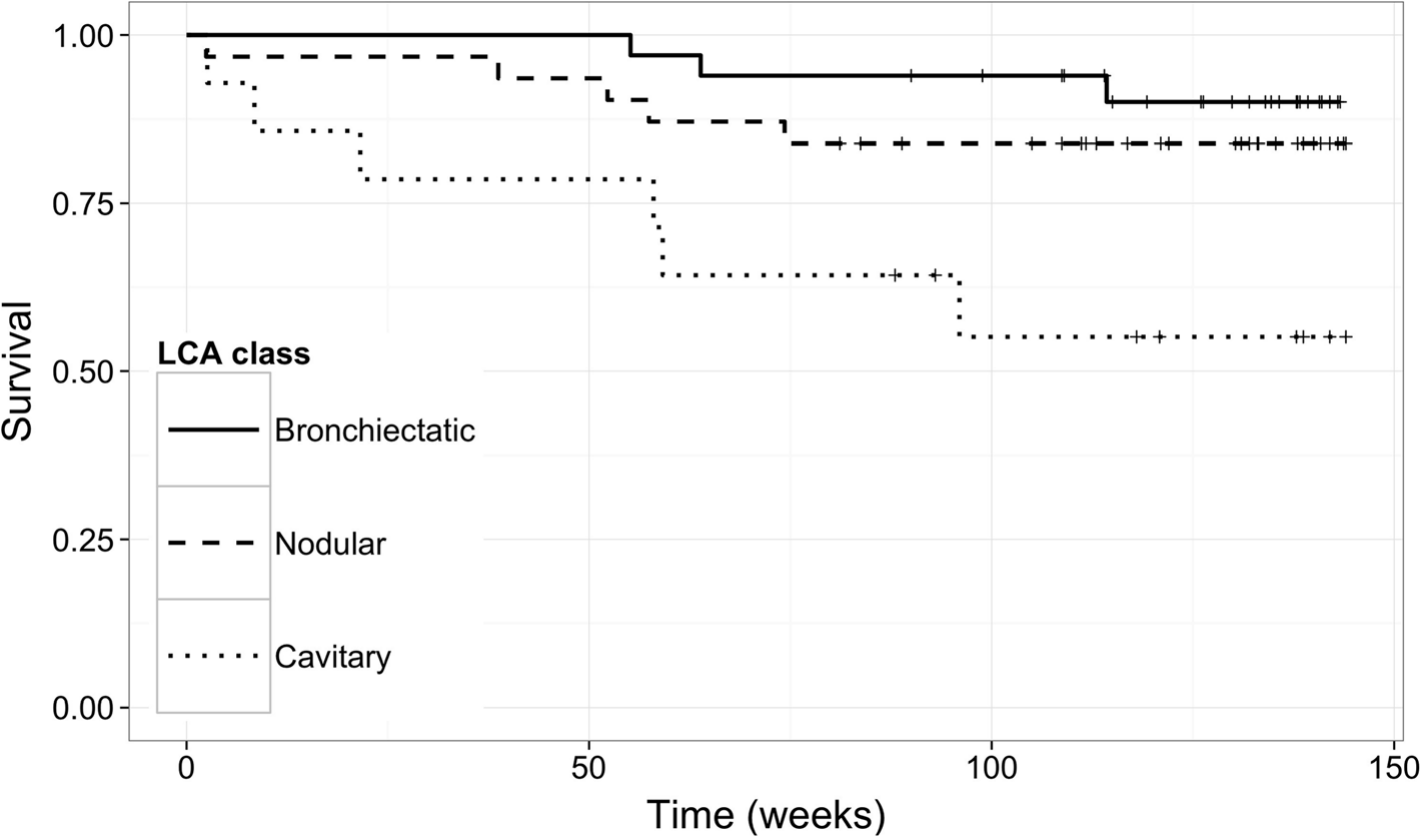
Kaplan-Meier survival curves for latent classes in the Royal Brompton cohort. X-axis = time (weeks), Y-axis = Proportion surviving, lines = latent classes

Survival data were available for 78 subjects with a median follow-up time of 126 weeks. There were significant differences in survival seen between groups, with the Cavitary group showing the highest mortality of 42.9% compared with the bronchietatic (9.1%) and Nodular (16.1%) groups (logrank test *P* = 0.011, Fig. 3).

### Replication cohort

CT scans from 62 patients were available from Papworth Hospital. Demographic details of the cohort are given in Additional file 1: Table S4 of the supplementary material. LCA identified three groups using the AIC (see Additional file 1: Figure S1). Class 1 comprised 8 (12.9%) subjects, class 2 comprised 19 (30.6%) subjects and class 3 comprised 35 (56.5%) subjects. The CT features of each class showed high similarity to the Brompton cohort (Fig. 1).

Class 1 corresponded to the Cavitary group with universally severe bronchiectasis which was highly extensive in 74.6%. Aspergillomas were present in 35.7% and the majority had severe cavitation (71.9%) although this was less than the universal presence seen in the Brompton cohort. Another point of difference was the high level of severe tree-in-bud change, seen in 87% but not seen in the Brompton cohort. Class 2 corresponded to the Nodular group, with generally mild changes except cavitating nodules which were present in 14.9% and severe cavitation in 20%. In contrast to the Brompton cohort, aspergillomas were present in 10% and a medium level of tree-in-bud changes in 40.2%. Class 3 corresponded to the Bronchiectatic group with all subjects having high severity bronchiectasis and 87.3% having highly extensive bronchiectasis. In contrast to the Brompton cohort the majority (90.5%) had a medium or high degree of tree-in-bud changes and no subjects had medium or high nodule scores compared with 12% in the Brompton cohort. Similarly, the vast majority had no cavitating nodules or severe cavitation, and aspergillomas were not seen. As with the Brompton cohort, class 1 showed the highest composite CT scores (mean 3.95), class 2 the lowest (mean 0.97) and class 3 intermediate values (mean 1.99). All differences were significant with *P* < 0.001 (Fig. 2b).

The mean age was youngest in class 2 and highest in the class 1, although this difference was not significant. There was significantly more underlying bronchiectasis in class 3. There was significantly more MAC (87.5%) in class 1 compared to other classes, whereas in the corresponding Cavitary group from the Brompton cohort this species accounted for only 50% of subjects.

### Subgroup analysis of subjects with bronchiectasis

A subgroup analysis was performed restricted to subjects with an underlying diagnosis of bronchiectasis. In the Brompton cohort (*N* = 43), two classes were identified by LCA representing 58 and 42% of the group (Additional file 1: Fig. S2). The first class was characterised by bronchiectasis of predominantly low severity and an absence of consolidation, severe cavitation or aspergillomas. It contained all 13 subjects in the Nodular subgroup of the full analysis, none from the Cavitary subgroup, and 12 subjects from the Bronchiectatic subgroup (see Additional file 1: Table S5 of the supplementary material). The second class was characterised by extensive and severe bronchiectasis, with severe cavitation in 30% and aspergillomas in 24%. This class contained all 5 subjects from the Cavitary subgroup of the full analysis, no subjects from the Nodular subgroup and 13 subjects from the Bronchiectatic subgroup.

In the Papworth cohort (*N* = 45) three classes were identified representing 62, 21 and 16% of the group. The features of these groups (Additional file 1: Fig. S3) closely resembled those found in the larger cohort (Fig. 1), corresponding to the ‘Bronchiectatic’, ‘Nodular’ and ‘Cavitary’ subgroups respectively. When compared with the original classification, 96% of subjects remained in the same subgroup (see Additional file 1: Table S5 of the supplementary material).

## Discussion

In this study, LCA identified three groups with distinct radiological characteristics in two independent cohorts of subjects with NTM-pulmonary disease. The finding of compatible radiological changes is a cornerstone of the diagnosis of NTM-pulmonary disease [5]. Historically the radiological pattern associated with NTM-pulmonary disease was one of cavitation similar to tuberculosis, typically seen in older males with underlying lung disease such as emphysema [18]. In the later twentieth century the first reports were published of a pattern of disease characterised by nodules and bronchiectasis which in contrast occurred in those with no underlying lung disease and was more common in women [19, 20]. The differentiation between the former ‘fibrocavitary’ disease and latter ‘nodular-bronchiectatic’ disease is of major clinical importance, as several studies have consistently demonstrated an association between fibrocavitary disease and mortality [6–8], disease progression [7] and treatment failure [21, 22]. This has been reflected in successive guidelines which recommend more aggressive treatment of fibrocavitary disease [5]. Other patterns of disease have been reported, in particular consolidation and infiltrative patterns have been associated with poor outcomes [9–11] however with no agreed consensus regarding definitions for these patterns, the findings remain unique to the individual studies. Even when an additional ‘consolidative’ category was used, one study found that 27% of subjects still did not clearly fit any category [3] and another was still unable to classify the radiological appearances of 229/481 subjects [9].

Our current study used a predefined scoring system to reduce subjectivity in assessing NTM related radiological changes, and latent class analysis as an unbiased method to identify groups sharing radiological characteristic without presupposing the existence of any specific patterns of disease.

The findings support the existence of fibrocavitary disease as a distinct pattern, identifying a group characterised by severe cavitation associated with markers of disease severity and a high mortality. There was a high prevalence of aspergilloma within this group, which has been associated with increased mortality in NTM-pulmonary disease [12].

Within the remaining majority of subjects, LCA identified two distinct subgroups, split evenly into those with very few radiological changes apart from nodules, and those with marked bronchiectasis. As may be expected, significantly more subjects in the latter Bronchiectatic group has pre-existing bronchiectasis, although this was also the case in over a third of those with ‘nodular’ disease. Interestingly despite having the best-preserved lung function and lowest symptom scores, there was no significant difference in mortality between the nodular and bronchiectatic groups. The duration of disease was significantly shorter in the nodular group, raising the possibility that this represents early disease which may progress to Cavitary or Bronchiectatic forms. A pattern of nodules and bronchiectasis have been associated with *M. avium* complex infection in a single study [23] however there were no differences in species prevalence between groups.

The validation cohort confirms the presence of three subgroups whose radiological characteristics shared many similarities with the Brompton cohort. There was a smaller group characterised by high composite CT scores with severe cavitation, the common presence of aspergillomas and severe and extensive bronchiectasis, corresponding to the Cavitary group. The other subjects were divided into those dominated by severe and extensive bronchiectasis without nodules, cavitation or aspergilloma (corresponding to the Bronchiectatic group) and those with low composite CT scores and low scores for most CT features (corresponding to the Nodular group). A higher proportion of subjects fell into the Bronchiectatic group, likely due to the higher overall prevalence of underlying bronchiectasis in this cohort.

The only major difference to the Brompton cohort was the higher prevalence of tree-in-bud changes, which were moderate or severe in 76% compared with 17%. The prevalence was higher in all three disease subgroups and does not appear to be due to the increased prevalence of underlying bronchiectasis. As the scoring of the validation cohort was performed separately to the Brompton cohort observer bias may be responsible for this difference, although this was performed by the same individual, alternatively it may be that differences in treatment, coinfection, or other clinical factors between centres is responsible. There was also more severe cavitation and aspergilloma in the nodular Papworth group compared with the Brompton, however only four subjects accounted for this difference.

In contrast to other studies mentioned previously, the presence of a separate consolidative pattern of NTM-pulmonary disease was not seen in this study. The vast majority of subjects in both cohorts had only mild consolidation, although this feature was slightly more prominent in the Cavitary group. Only one subject had consolidation and no other feature of NTM disease. Our findings may in part be explained by the absence of immunocompromised or critically ill subjects from our cohorts. When the analysis was restricted to subjects with known underlying bronchiectasis only two subgroups were identified, with the Bronchiectatic group being split in half dividing the cohort into groups more consistent with the conventional ‘fibrocavitary’ and ‘nodular-bronchiectatic’ patterns. However, in the validation cohort the three subgroups were still clearly identified.

The principle limitation of the study is the heterogeneous nature of the cohort, comprising subjects with differing underlying respiratory diseases, NTM species, antibiotic treatments and infecting co-pathogens. Nevertheless the findings were replicable in an independent cohort suggesting they are not merely related to the specific case mix at a single centre. Furthermore, such a mix of subjects is representative of real-life clinical practice, although as both cohorts come from tertiary centres the proportion of subjects with severe bronchiectasis and aspergillomas may be higher than the wider population of individuals with NTM-pulmonary disease. A number of subjects had previously undergone HRCT and declined further imaging, therefore in some subjects the collected clinical data may not reflect the point in time when the HRCT was performed and in 10% of cases more than a year had elapsed from imaging to study enrolment. A single radiologist was responsible for performing CT scoring and despite the use of an objective scoring system this remains a potential source of bias, although the scoring system was based on the Bhalla score [24] which has been shown to have low interobserver variability [25].

## Conclusions

This study provides validation of the existence of cavitary disease as a distinct phenotype of NTM-pulmonary disease associated with a poor prognosis. In addition they suggest that ‘nodular-bronchiectatic’ disease is formed of two separate groups with important radiological and clinical differences, which may possibly reflect differences in underlying lung disease or duration of infection. These data underline that fact that NTM-pulmonary disease is a heterogeneous condition and more precise phenotyping will be valuable in clinical decision making and stratification in clinical trials.

## Additional file

Additional file 1: Supplementary materials. (DOCX 1280 kb)

## Abbreviations

AIC: Aikake information criteria
ANOVA: Analysis of variance
BMI: Body mass index
COPD: Chronic obstructive pulmonary disease
CRP: C-reactive protein
CT: Computed tomography
FEV_1_: Forced expiratory volume in 1 s
FVC: Forced vital capacity
HIV: Human immunodeficiency virus
HRCT: High resolution computed tomography
LCA: Latent class analysis
MRC: Medical Research Council dyspnoea scale
NTM: Nontuberculous mycobacteria
SGRQ: St George’s Respiratory Questionnaire
TLCOc: Corrected transfer factor for carbon monoxide
