# Supplementary material for: Latent class analysis to define radiological subgroups in pulmonary nontuberculous mycobacterial disease
Source: BMC Pulm Med. 2018 Aug 31;18:145. doi: 10.1186/s12890-018-0675-8 (PMC6119278; doi:10.1186/s12890-018-0675-8)
Supplement: Supplementary file 1 — Supplementary materials. (DOCX 1280 kb) [file 12890_2018_675_MOESM1_ESM.docx]

**Latent Class Analysis to Define Radiological Subgroups in Pulmonary Nontuberculous Mycobacterial Disease**

Steven A. Cowman, Joseph Jacob, Sayed Obaidee, R. Andres Floto, Robert Wilson, Charles S. Haworth and Michael R. Loebinger.

**Supplementary Material**

**Recruitment**

Subjects were identified through a search of clinic letters, new referral letters and by notification from the Microbiology department of new mycobacterial smear and culture results during the study period. Exclusion criteria were: age < 18 years, pregnancy, active malignancy, active tuberculosis, cystic fibrosis and immunosuppression other than oral prednisolone.

Subjects were defined as having no underlying pulmonary disease if prior to their diagnosis with NTM-pulmonary disease they had never been diagnosed with a pulmonary disease.

|  | **Bronchiectasis extent** | **Bronchiectasis severity** | **Tree-in-bud opacification** | **Nodules** | **Cavitating nodules** | **Severe cavitation** | **Consolidation** | **Aspergilloma** |
| --- | --- | --- | --- | --- | --- | --- | --- | --- |
| **Right upper lobe** |  |  |  |  |  |  |  |  |
| **Left upper lobe** |  |  |  |  |  |  |  |  |
| **Right middle lobe** |  |  |  |  |  |  |  |  |
| **Lingula** |  |  |  |  |  |  |  |  |
| **Right lower lobe** |  |  |  |  |  |  |  |  |
| **Left lower lobe** |  |  |  |  |  |  |  |  |
| **Total** |  |  |  |  |  |  |  |  |

Table S1. CT scoring matrix. The features were scored as follows: Bronchiectasis extent: 0 = none; 1 = 1 bronchopulmonary segment, 2 = >1 bronchopulmonary segments, 3 = >2 bronchopulmonary segments; Bronchiectasis severity: 0 = none, 1 = mild (1-2x diameter of accompanying artery), 2 = severe (>2x diameter of accompanying artery); Tree-in-bud opacification : 0 = none, 1 = mild, 2= moderate, 3 = severe; Nodules: 0 = none, 1 = 1 nodule, 2 = 2-4 nodules, 3 = >4 nodules; Cavitating nodules: 0 = no, 1 = yes; Severe cavitation: 0 = no, 1 = yes; Consolidation: 0 = none, 1 = mild, 2 = moderate, 3 = severe; Aspergilloma: 0 = no, 1 = yes. Nodules were only counted if >5mm diameter, Severe cavitation was defined as the pattern of cavitation classically seen in tuberculosis. Adapted from Zoumot *et al.* [1]

| **Female sex** | 54 (63.5%) |
| --- | --- |
| **Age (years)** | 65.3 (±10.8) |
| **Smoking history** |  |
| Never smoker | 41 (48.2%) |
| Ex-smoker | 36 (42.4%) |
| Current smoker | 8 (9.4%) |
| **Ethnicity** |  |
| Asian | 6 (7.1%) |
| Black | 2 (2.4%) |
| Chinese | 2 (2.4%) |
| White | 75 (88.2%) |
| **Diagnosis** |  |
| Bronchiectasis | 43 (50.6%) |
| COPD | 30 (35.3%) |
| Other | 8 (9.4%) |
| No underlying lung disease | 14 (16.5%) |
| **Systemic corticosteroid use** | 16 (18.8%) |
| **Inhaled corticosteroid use** | 48 (56.5%) |
| **Oral antibiotic prophylaxis** | 22 (25.9%) |
| **Nebulised antibiotic prophylaxis** | 7 (8.2%) |
| **FEV1 (% predicted)** | 61.7 (±24.3) |
| **FVC (% predicted)** | 91.0 (±20.7) |
| **TLCOc (% predicted)** | 59.3 (±23.0) |
| **BMI (kg/m^2^)** | 21.8 (±4.1) |
| **SGRQ total score** | 45.8 (±24.1) |
| **Chronic *Pseudomonas* infection** | 10 (11.8%) |
| **Mycetoma** | 5 (5.9%) |
| **Semi-invasive Aspergillus disease** | 3 (3.5%) |
| **Dominant NTM species** |  |
| *M. abscessus* | 14 (16.5%) |
| *M. avium complex* | 44 (51.8%) |
| *M. kansasii* | 9 (10.6%) |
| *M. xenopi* | 11 (12.9%) |
| Other | 7 (8.2%) |
| **Interval since diagnosis (years)** | 2.9 (±3.8) |
| **Age at diagnosis (years)** | 62.4 (±11.3) |
| **Currently receiving anti-mycobacterial treatment** | 18 (21.2%) |
| **Ever received anti-mycobacterial treatment** | 37 (43.5%) |

**Table S2.** Clinical characteristics of the Royal Brompton cohort.

| **CT feature** | **Score** | **Brompton**  N = 85 | **Papworth**  N = 62 |
| --- | --- | --- | --- |
| **Bronchiectasis extent** | Low | 26 (30.6%) | 17 (27.4%) |
|  | Medium | 26 (30.6%) | 29 (46.8%) |
|  | High | 33 (38.8%) | 16 (25.8%) |
| **Bronchiectasis severity** | Low | 54 (63.5%) | 24 (38.7%) |
|  | High | 31 (36.5%) | 38 (61.3%) |
| **Tree-in-bud opacification** | Low | 71 (83.5%) | 15 (24.2%) |
|  | Medium | 12 (14.1%) | 32 (51.6%) |
|  | High | 2 (2.4%) | 15 (24.2%) |
| **Nodules** | Low | 74 (87.1%) | 57 (91.9%) |
|  | Medium | 11 (12.9%) | 3 (4.8%) |
|  | High | 0 (0%) | 2 (3.2%) |
| **Cavitating nodules** | Absent | 69 (81.2%) | 53 (85.5%) |
|  | Present | 16 (18.8%) | 9 (14.5%) |
| **Severe cavitation** | Absent | 69 (81.2%) | 51 (82.3%) |
|  | Present | 16 (18.8%) | 11 (17.7%) |
| **Consolidation** | Low | 79 (92.9%) | 59 (95.2%) |
|  | Medium | 6 (7.1%) | 2 (3.2%) |
|  | High | 0 (0%) | 1 (1.6%) |
| **Aspergilloma** | Absent | 77 (90.6%) | 52 (83.9%) |
|  | Present | 8 (9.4%) | 10 (16.1%) |

**Table S3.** Scores of individual CT features in the Brompton and Papworth cohorts. Figures indicate numbers of subjects.


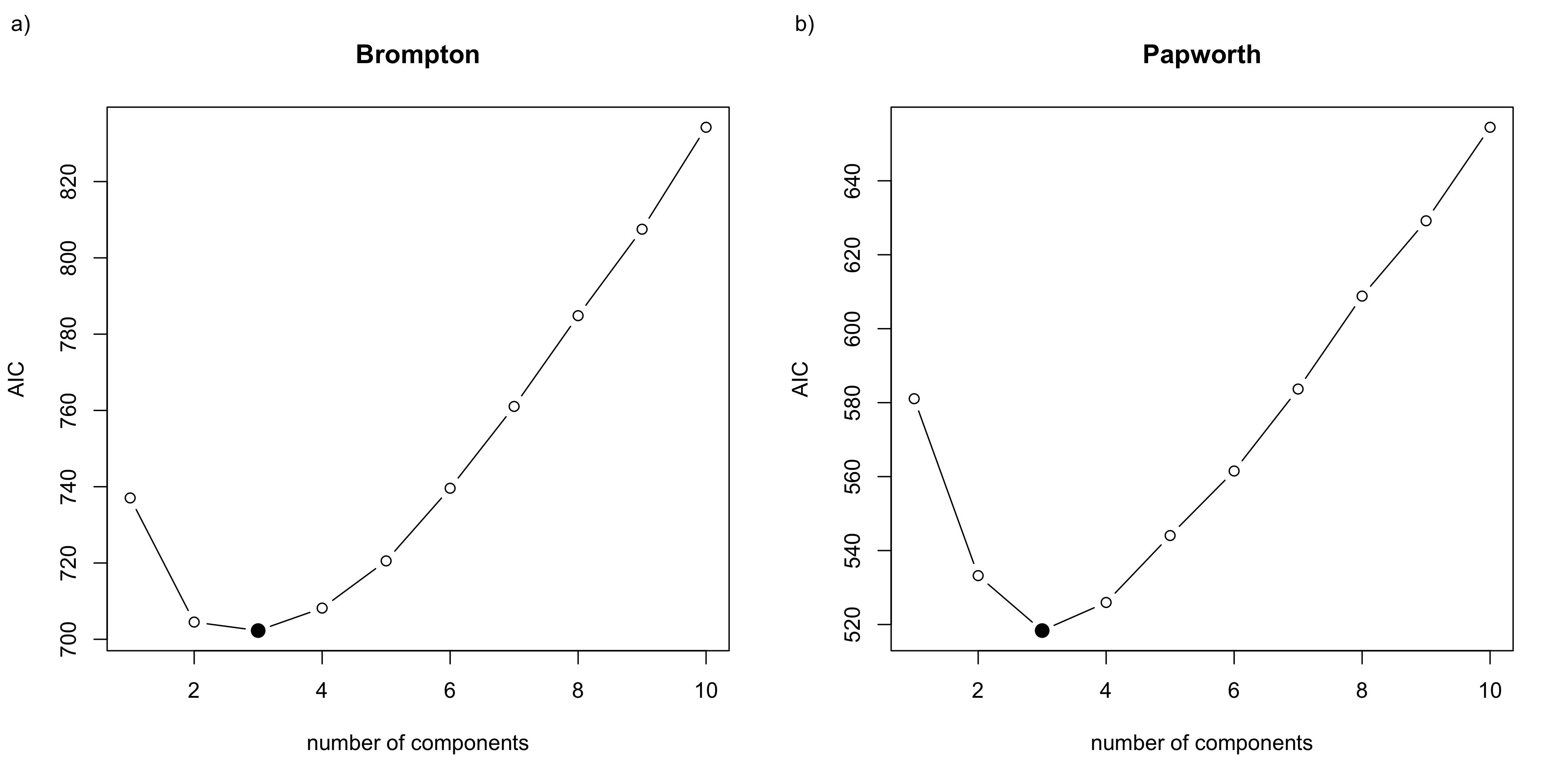


**Figure S1.** Identification of the optimum number of groups in a) the Royal Brompton cohort and b) the Papworth cohort. AIC = Aikake information criteria.

|  | **Total** | **Class** | | | ***P* value** |
| --- | --- | --- | --- | --- | --- |
|  |  | **1** | **2** | **3** |  |
|  | N = 63 | N = 8 | N = 19 | N = 35 |  |
| **Female Sex** | 36 (58.1%) | 7 (87.5%) | 8 (42.1%) | 21 (60.0%) | 0.099 |
| **Age (years)** | 68.3 (±14.8) | 71.8 (±11.3) | 64.7 (±17.8) | 69.4 (±13.8) | 0.432 |
| **Diagnosis** |  |  |  |  | **0.033** |
| Bronchiectasis | 45 (72.6%) | 5 (62.5%) | 10 (52.6%) | 30 (85.7%) |  |
| COPD | 7 (11.3%) | 1 (12.5%) | 4 (21.1%) | 2 (5.7%) |  |
| Other | 6 (9.7%) | 0 (0.0%) | 4 (21.1%) | 2 (5.7%) |  |
| No underlying lung disease | 4 (6.5%) | 2 (25.0%) | 1 (5.3%) | 1 (2.9%) |  |
| **NTM species** |  |  |  |  | 0.880 |
| *M. avium* complex | 45 (72.6%) | 7 (87.5%) | 13 (68.4%) | 25 (71.4%) |  |
| *M. abscessus* | 8 (12.9%) | 1 (12.5%) | 2 (10.5%) | 5 (14.3%) |  |
| *M. kansasii* | 1 (1.6%) | 0 (0.0%) | 0 (0.0%) | 1 (2.9%) |  |
| *M. xenopi* | 1 (1.6%) | 0 (0.0%) | 1 (5.3%) | 0 (0.0%) |  |
| Other species | 7 (11.3%) | 0 (0.0%) | 3 (15.8%) | 4 (11.4%) |  |

**Table S4.** Clinical characteristics of the Papworth cohort.

**
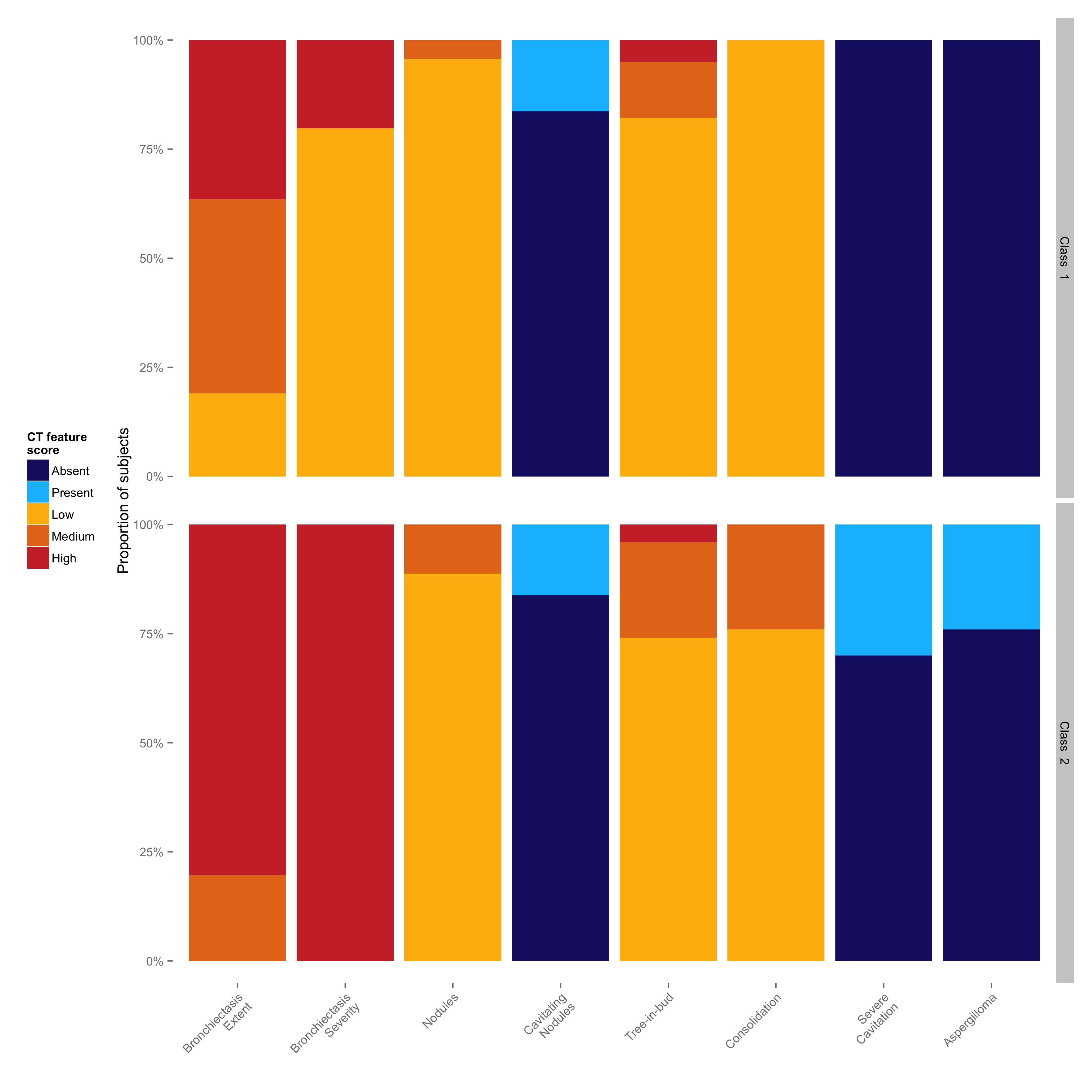
**

**Figure S2. CT features of latent classes identified in subjects with bronchiectasis in the Royal Brompton cohort.** The individual CT features used in the NTM scoring system are shown on the x-axes and the proportion of subjects on the y-axes. Colours represent the severity (low, medium or high), or the presence or absence of the CT feature.

| a) |  | Original Analysis | | |
| --- | --- | --- | --- | --- |
|  |  | Cavitary | Nodular | Bronchiectatic |
| Bronchiectasis only analysis | Class 1 | 0 | 13 | 12 |
|  | Class 2 | 5 | 0 | 13 |
|  |  |  |  |  |
| b) |  | Original Analysis | | |
|  |  | Cavitary | Nodular | Bronchiectatic |
| Bronchiectasis only analysis | Class 1 | 0 | 0 | 29 |
|  | Class 2 | 0 | 9 | 0 |
|  | Class 3 | 5 | 1 | 1 |

**Table S5. Comparison of latent class membership in bronchiectasis subgroup analysis versus original analysis of the a) Brompton and b) Papworth cohorts.**


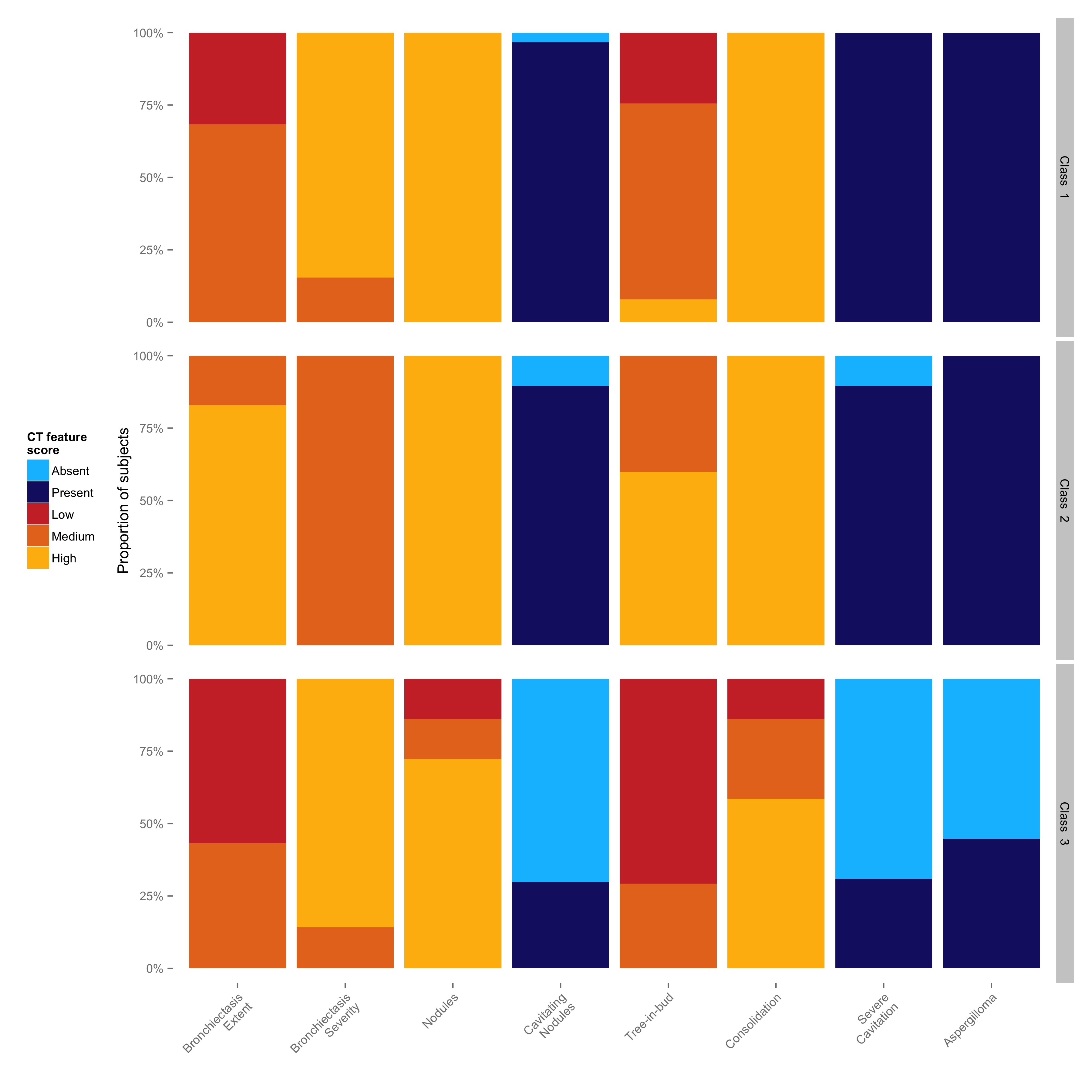


**Figure S3. CT features of latent classes identified in subjects with bronchiectasis in the Papworth cohort.** The individual CT features used in the NTM scoring system are shown on the x-axes and the proportion of subjects on the y-axes. Colours represent the severity (low, medium or high), or the presence or absence of the CT feature.

**Supplementary References**

1. Zoumot Z, Boutou AK, Gill SS, van Zeller M, Hansell DM, Wells AU, et al. Mycobacterium avium complex infection in non-cystic fibrosis bronchiectasis. Respirology. 2014;19:714–22.
